# Integrated Management and Visualization of Electronic Tag Data with Tagbase

**DOI:** 10.1371/journal.pone.0021810

**Published:** 2011-07-05

**Authors:** Chi Hin Lam, Vardis M. Tsontos

**Affiliations:** 1 Marine Environmental Biology, Department of Biological Sciences, University of Southern California, Los Angeles, California, United States of America; 2 Fisheries Resources Division, NOAA/NMFS Southwest Fisheries Science Center, La Jolla, California, United States of America; University of California Davis, United States of America

## Abstract

Electronic tags have been used widely for more than a decade in studies of diverse marine species. However, despite significant investment in tagging programs and hardware, data management aspects have received insufficient attention, leaving researchers without a comprehensive toolset to manage their data easily. The growing volume of these data holdings, the large diversity of tag types and data formats, and the general lack of data management resources are not only complicating integration and synthesis of electronic tagging data in support of resource management applications but potentially threatening the integrity and longer-term access to these valuable datasets. To address this critical gap, Tagbase has been developed as a well-rounded, yet accessible data management solution for electronic tagging applications. It is based on a unified relational model that accommodates a suite of manufacturer tag data formats in addition to deployment metadata and reprocessed geopositions. Tagbase includes an integrated set of tools for importing tag datasets into the system effortlessly, and provides reporting utilities to interactively view standard outputs in graphical and tabular form. Data from the system can also be easily exported or dynamically coupled to GIS and other analysis packages. Tagbase is scalable and has been ported to a range of database management systems to support the needs of the tagging community, from individual investigators to large scale tagging programs. Tagbase represents a mature initiative with users at several institutions involved in marine electronic tagging research.

## Introduction

Electronic tagging studies are providing fundamental insights into the spatial ecology of marine species [1], [2], [3], also in support of fisheries assessment and ecosystem-based management efforts [4], [5], [6]. Tags are applied to study a wide range of taxa from tunas [7], [8], [9], [10], sharks [11], [12], billfishes [13], [14], [15], turtles [16], [17], squids [18] to birds [19], [20]. This proliferation of tagging programs and tag deployments generates ever-increasing volumes of data on the movement dynamics, physiology and habitat preferences of pelagics. To ensure ease of access for synthesis [21] and the legacy of these research programs [22], the effective management of tag data is critically important and currently an issue.

Software tools from tag manufacturers are designed principally for processing individual datasets, and understandably focus on their own products. While these are suited to analyzing single tag datasets, researchers typically utilize tags from various manufacturers and deal with numerous tags from multi-year studies. In the absence of accessible database solutions dealing with the complexities of tagging data generically, the logistics of tag management is proving a major impediment to researchers. This impact may be less severely felt by groups possessing informatics infrastructure and support (e.g. Tagging of Pacific Pelagics [23] or OBIS-SEAMAP [24]) However, many researchers are at best either embarking on parallel development of tag databases often without the requisite IT expertise or more typically attempting to deal with extensive archives of heterogeneous native flat files within software not designed for data management, such as familiar spreadsheet environments. This not only consumes resources and renders analyses inefficient to conduct but ultimately may compromise access to and the integrity of tagging datasets longer term [25], [26], [27].

Some efforts have been made to address issues of tag data management for marine species through systems such as the web-based Satellite Tracking and Analysis Tool [28], and CSIRO's institutional tagging database [29]. These tools differ in design and capability (Table 1), but also in terms of their portability and accessibility which complicate their adoption by the broader tagging community. For example, STAT tool is easy to use via its web interface but is primarily designed to work only with Argos and GPS positioning data. Alternatively, the CSIRO Oracle-based system supports multiple tag types but is not readily transferable and requires dedicated data management expertise and infrastructure that are typically unavailable.

**Table 1:** Comparison matrix of database management tools for electronic tag data in marine applications.

Tool	User interface	Backend database	Data connectivity	Tag type				Argos fetching	Mapping	Cost	Website	References
				*Acoustic*	*Archival*	*Popup archival*	*Telemetry (GPS/ Argos)*					
Satellite Tracking and Analysis Tool (STAT)	Web browser	MySQL	Only via text file export	X	X	X	✓	✓	✓	Free; web service	www.seaturtle.org/stat	[28]
CSIRO database	Microsoft Access/ Custom webpage	Oracle	Open Database Connectivity (ODBC)	✓	✓	✓	✓	✓	✓	Open to collaboration	www.marine.csiro.au/cgi-bin/ags/etss_access_public.pl	[29]
Tagbase	Microsoft Access	Microsoft Access / SQL Server	Open Database Connectivity (ODBC)	X	✓	✓	✓	X	✓	Free; open source	code.google.com/p/tagbasewww.tagbase.org	this paper

**Figure 1:**
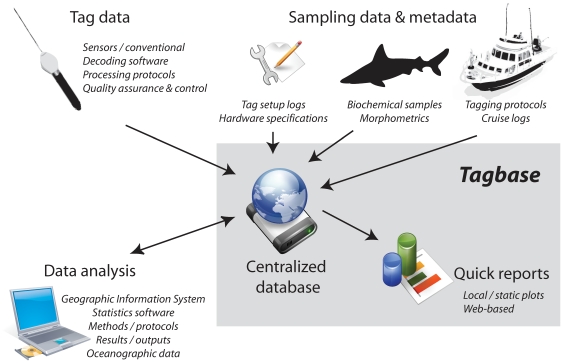
Aspects of electronic tag data processing workflow. Within this framework, Tagbase provides a comprehensive database management solution with built-in graphical capability to quickly visualize tag data (gray area).

Tagbase addresses these critical constraints by providing an accessible, stand-alone tag data management system with an integrated set of analysis tools aimed particularly at the individual tag researcher or research group level (Figure 1). Key features include: 1) rapid assimilation of tag data from multiple tag types with minimal setup, 2) a robust, generalized, and scalable tag data management platform that requires no user technical expertise or intervention, 3) a well-rounded set of integrated tools for visualizing and summarizing data in standard ways, and 4) online support at Tagbase.org and a community-driven, open-source development model. Tagbase aims to empower researchers to efficiently work with their data directly. This is achieved by focusing the development on the majority of available tag types, and by leveraging tools compatible with the widely used Microsoft (MS) Office suite. Tagbase features automated bulk import of processed files (Tables 1 & 2), but relies on users to perform beforehand the necessary processing with manufacturer software after tag reporting or retrieval and recommended quality control screening. Essentially, Tagbase jumpstarts tag data management by providing a well-rounded, flexible, user-friendly database solution for electronic tagging applications. Its extensible, open architecture facilitates maintainability and porting to enterprise database systems as necessary. Future developments of Tagbase will support acoustic tags and open-source software.

**Table 2:** Supported input formats of Tagbase.

Tag manufacturer	Tag types	Supported processed files from software	Website
Lotek Wireless	Archival	Viewer2000	www.lotek.com/downloads.htm
Microwave Telemetry	Popup archival / recovered popup	Outputs from proprietary decoding service	www.microwavetelemetry.com
Wildlife Computers	Archival, popup archival, SPOT, Argos Fast-GPS	WC-AMP, HexDecode, WC-DAP 2.0 & 3.0, GPE version 1 & 2	www.wildlifecomputers.com/downloads.aspx

## Methods

Tagbase is currently implemented in relational databases running on MS Windows operating systems. Tagbase was initially developed within MS Access because of its general availability and familiarity. Furthermore, Access's current 2-GB size limit has not proved to be an impediment to the adoption of Tagbase by the smaller research groups it was targeted for, particularly those not working extensively with archival tags. However, for secure, network-based management of tagging data of larger electronic data archives, an SQL Server implementation of Tagbase exists. This is an enterprise solution that can host large-scale electronic tag datasets in the centralized SQL Server back-end database while allowing users to seamlessly interface via Tagbase Access clients on the front-end over a LAN. This approach leverages existing Tagbase functionalities in Access to interactively import/export datasets, view metadata and plot data via a dynamic Open Database Connectivity (ODBC) connection to SQL Server. Such client-server architecture also sets the stage for future development of browser-based access through a Web-form interface. The design is also sufficiently generic to allow future porting of the Tagbase back-end to other proprietary databases, such as Oracle, or open-source industrial strength systems such as Postgre SQL.

### Relational model

Tagbase implements a unified relational model for the management of electronic tagging data. Its normalized design encapsulates and integrates in a generalized yet parsimonious manner the range of data outputs from various tag models and manufacturers, together also with critical deployment metadata and information from geolocation post-processing. The relational design: 1) compactly and accurately reflects the fundamental logical organization of information in a way that is easily understood; 2) uses appropriate data structures and validation controls to ensure data integrity; 3) employs normalization to optimize storage, querying and maintainability of the database; 4) implements indexing for efficient access.

**Figure 2:**
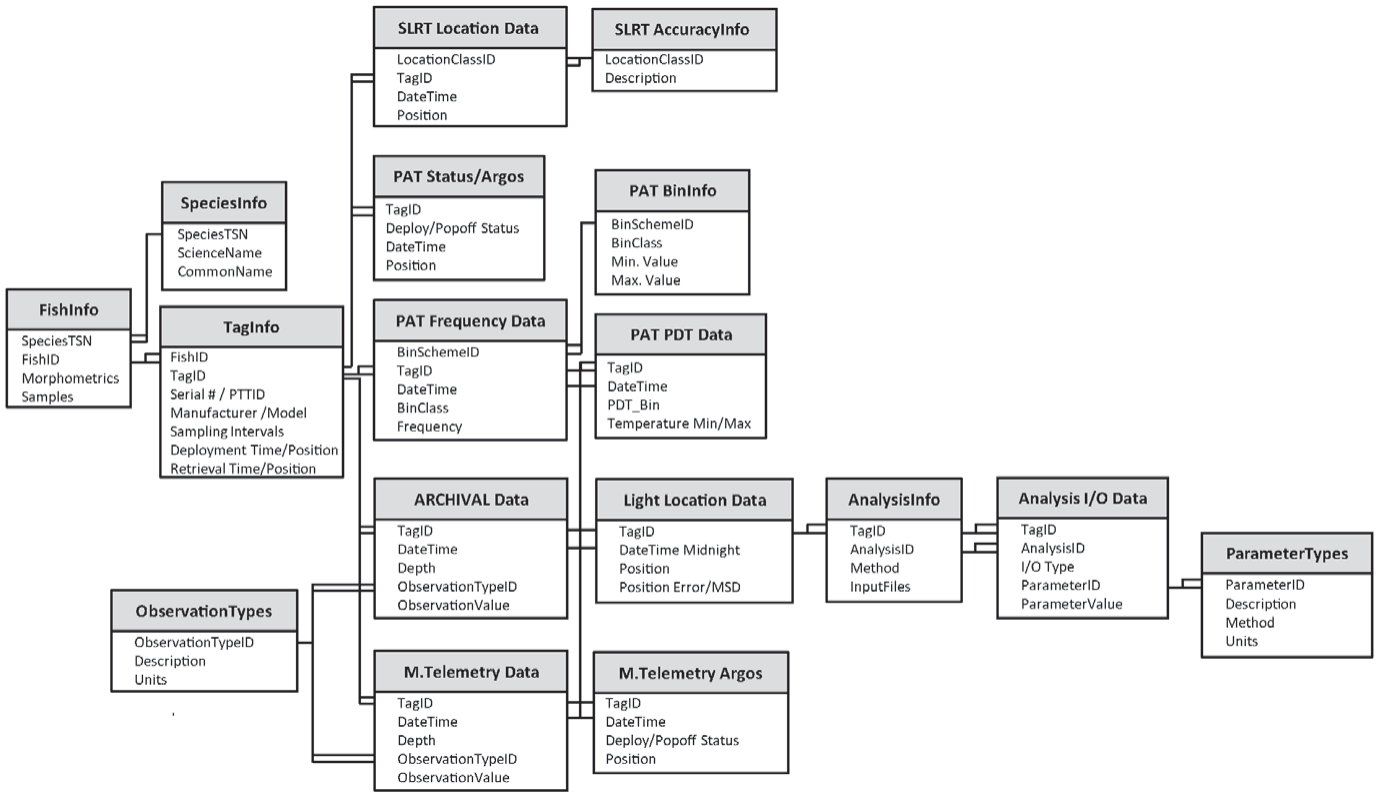
Entity-Relationship diagram summarizing the Tagbase relational data model. Tables, composed of thematically and structurally distinct sets of information, appear as boxes with descriptive table name headers and a list of constituent fields. Relationships between tables in this information hierarchy are shown as lines linking primary and foreign key fields in adjacent tables. One-to-one relationships are illustrated as single terminal lines (−). One-to-many relationships between key fields are represented as double ( = ) and single lines on each terminus, linking multiple records in the child table to single rows in the parent table respectively.

Tagbase's relational model is shown schematically in Figure 2 and is characterized by hierarchically related tables, grouped according to the basic type of data they hold. The **FishInfo** table holds species code and other information (e.g. morphometrics) describing each tagged animal. This is related to the **TagInfo** table which contains key information about tags deployed on individuals (e.g. model, serial number, deployment and retrieval locations and times). Linkage always is via an *ID* field, which is a unique numerical identifier assigned to each record in the parent table and present in the child table as a foreign key. The one-to-many relationship between these tables accommodates scenarios where either single or multiple tags are deployed on individual animals.

The adjacent block of related lower level tables holds the detailed electronic tagging data themselves. Data associated with different tag types are associated with distinct table blocks (Figure 2).

Satellite-linked radio telemetry (SLRT) tag data, which are animal location series from positional satellites (e.g. Argos), are maintained in the **SLRTLocation** table, with lookups to transmission accuracy descriptions in the **SLRTAccuracyInfo** code table. Transmitted popup archival tag (PAT) data are aggregate summaries of raw archival series maintained within the following four tables: **PAT_Frequency** holds both time-at-temperature and time-at-depth series data, any arbitrary binning scheme being accommodated as a result of the table's normalized design and linkage to the **PAT_BinInfo** table that contains details of user defined class intervals; **PAT_PDT** contains the series of minimum and maximum temperature data within dynamically varying depth intervals; and the **PAT_Status** table contains Argos positions recorded once the tag detaches from the animal and transmits at the surface. Detailed time series from archival tags and recovered PAT tags reside within the **Archival** table with light-based estimates of animal positions in the **LightLocation** table. Normalization of the former by inclusion of an *ObservationTypeID* field rather than typical columnar structure for observed variables allows this table to flexibly accommodate data from multiple manufacturers and is maintainable since it can accept any arbitrary number of observation types from archival tag sensors. Descriptions of archival observation variables are available via lookups on the **ObservationTypes** table, which is also referenced by the table holding the core **MicrowaveTelemetry** data. Popoff locations for these tags are maintained in the **M.TelemetryArgos** table.

The final set of tables is the **Analysis** table block, which holds both results and metadata from geolocation post-processing. Several geolocation algorithms exist and are being used by the tagging community, each with their own particular sets of parameters and output formats for estimated track positions. Management and linkage of analysis results and parameter metadata to source tag datasets have posed significant challenges to researchers prior to Tagbase. The generalized design of Tagbase's **Analysis** tables accommodates metadata and outputs from any of the currently used algorithms in addition to variants of these that are likely to arise in future. The design also handles scenarios where potentially multiple geo-positional analyses are conducted using different methods or the same algorithm with different parameter selections are applied. It also allows estimated positions to be traced back to and matched against any other type of related data maintained within Tagbase, including manufacturer light-based positional estimates or GPS or Argos positions from telemetry tags in the case of double tagging experiments.

### Import capabilities

Tagbase provides users with an interactive form interface to import data effortlessly into the database. All the complex mechanics of transforming diverse, heterogeneously structured tagging data from native manufacturer formats (Table 2) are all automated and handled by Tagbase behind the scenes.

**Figure 3:**
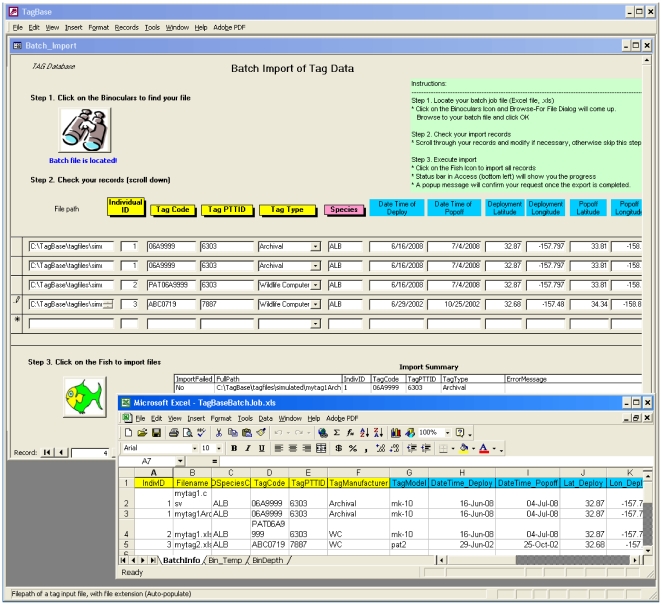
Automated importation of various tag data files via a simple import form in Tagbase. A job file (bottom window), which contains the file locations and tag metadata, is first prepared and then loaded into Tagbase (top window). Import is then initiated with a click of a button in the form.

The import process in Tagbase is straightforward and is initiated by first filling out an import job file with key metadata such as the source file name and path, the tag type, tag deployment and retrieval information, and other tag model specific information (Figure 3). Both individual and multiple tag datasets can be batch-imported in a single job. Next, the user runs the import form in Tagbase and points to the job file to display the metadata for any final edits before clicking a button to import all specified tag datasets (Figure 3). Tagbase automatically undertakes restructuring of data for inclusion into tables via a series of stored queries and macros. This entire process is efficient, with a run time of a minute or two per archival tag (e.g. ∼50 megabytes of data).

### Export support

**Figure 4:**
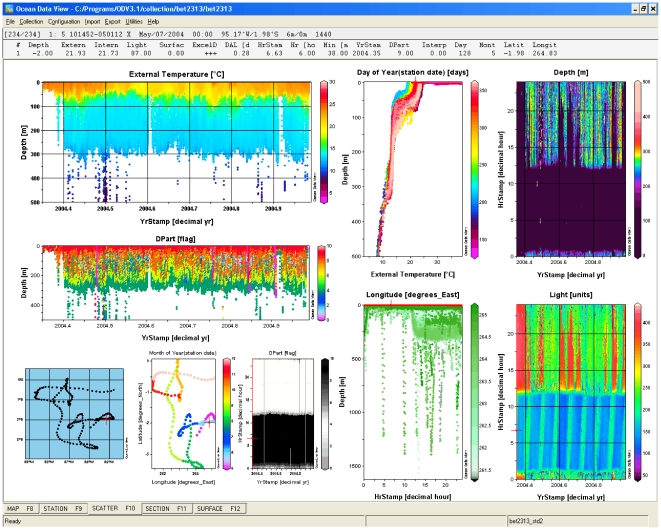
Tagbase's advanced ODV visualization capability illustrating a bigeye tuna archival dataset. Ocean Data View (ODV, [30]) provides flexible plotting and interactive display of tag data. Multiple plot windows can be set up easily with simple user interfaces.

Various widely used third party analysis packages for tag data geolocation and visualization require datasets to be formatted in very specific ways for usage. Tagbase provides standard tools for exporting data as delimited text files (.csv) for those external packages most frequently used by tag researchers and whose formats are more complicated and most difficult to reproduce (Table 3). While Tagbase's integrated plot forms allow a range of standard visualizations to be produced interactively, the MS Graph control component used does not allow for more advanced visualization. This is achieved indirectly by exporting to a specialized package, Ocean Data View (ODV [30]) via a Tagbase form linked to a stored query procedure that packages the data appropriately (Table 3; Figure 4). Estimating positions for a tag based on light level and other oceanographic parameters, often referred to as geolocation, is another frequent operation that a researcher will need to perform on tag data. Such statistical analyses are conducted in other software, and Tagbase supports export to the open-source R packages like Kftrack [31], Ukfsst [32], [33], and Trackit [34], [35] widely used to estimate track positions (Table 3). To facilitate usage of the more advanced, Trackit geolocation package [34], scripts for running this package in R are also available via Tagbase.

**Table 3:** Supported output formats of Tagbase.

Software	Purpose	Website	References
Kftrack (R)	Geolocation	www.soest.hawaii.edu/tag-data/software	[31]
kfsst/ ukfsst (R)	Gelocation	www.soest.hawaii.edu/tag-data/software	[32], [33]
Trackit (R)	Gelocation	www.soest.hawaii.edu/tag-data/software	[34], [35]
Ocean Data View	Analysis and visualization	odv.awi.de	[30]
Shapefiles for ArcGIS	Geographic Information System	www.esri.com	---

### Forms for Data Visualization

**Figure 5:**
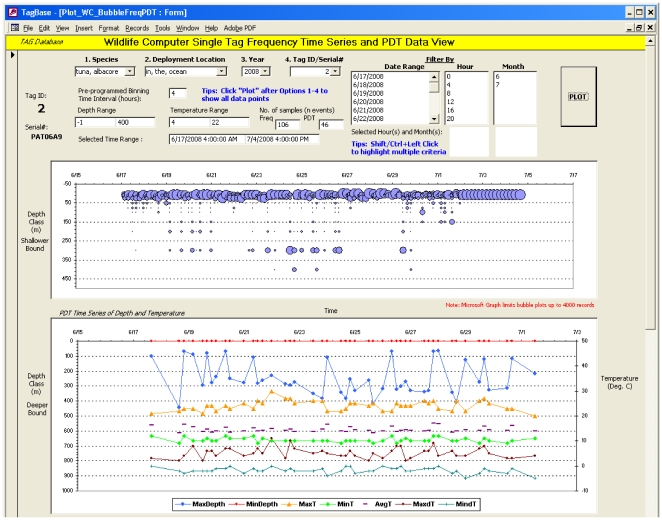
Rapid visualization of tag data in Tagbase. Tagbase forms provide a user-friendly interface for rapidly sub-setting data via interactive controls to produce standard reports as both tabular and graphical outputs. Controls at the top of the form allow selection of source data from single or multiple tags or dates. Wildlife Computers PAT time-at-depth series (bubble plots) and PDT time series (line plots) are shown here.

Tagbase includes a range of forms that allow rapid summarization of either individual or aggregate tag datasets, the intent being that users are able to efficiently explore their data and produce standard outputs in both tabular and graphical form within the Tagbase environment. A representative example of these is shown in Figure 5, although plot forms are available for all tag and data types, also incorporating day-night and lunar phase information. In all cases, pull-down lists and other interactive controls at the top of the form allow users to dynamically select and subset data for display. Embedded plot objects offer MS Excel-style graphing capability with interactive formatting and access to underlying source data that can be pasted into external applications via the clipboard. More advanced users can design additional displays leveraging Tagbase's infrastructure and Visual Basic codebase to customize the application according to their particular needs.

### Interactive Mapping

Mapping is an important part of tag data analyses and integral to Tagbase. Tagbase achieves this natively without requiring export to external geographical information system (GIS) software or mapping web services. Mapping functionality is mediated by the MapWindow ActiveX form control (Geospatial Software Lab, Idaho State University), an open-source GIS component (Table 4) with functionality including zooming and panning, layering, raster image display, and shapefile generation, attribute filtering, labeling and coloring.

**Table 4:** Free utilities and plug-ins used in Tagbase.

Tool	Version	Function	Source
MapWinGis ActiveX control	47SRa	Open-source mapping control and library	www.mapwindow.org/downloads/index.php?show_details=2 mapwingis.codeplex.com
TortoiseSVN	1.6.12	Version management and backup	tortoisesvn.tigris.org
vcredist_x86.exe	x86	Microsoft Visual C++ 2008 runtime libraries for a computer that does not have Visual C++ 2008 installed	www.microsoft.com/downloads/en/details.aspx?familyid=A5C84275-3B97-4AB7-A40D-3802B2AF5FC2&displaylang=en
wget.exe	1.11.4	HTTP/ FTP download	www.gnu.org/software/wgetusers.ugent.be/∼bpuype/wget
XtrFun.dll	3.1.4.0	Curve-fitting, interpolation of 2-dimensional and 3-dimensional data	www.xlxtrfun.com/XlXtrFun/XlXtrFun.htm

Tagbase's mapping features allow both visualization and dynamic interaction with tag data in a spatial context. The tool also facilitates the production of shapefiles from geo-referenced track data and associated attribute information, such as tag or animal metadata or recorded tag data (e.g. daily maximum diving depth) via simple queries (Table 3). Once tracks are mapped, users can access the detailed underlying tag observations interactively by clicking on particular points of interest or windowing to select collections of points. Data are then instantly assembled from source tables in Tagbase and used to populate appropriate standard plot forms. Such geographical selection and data retrieval allows a highly efficient and integrated way to visualize tag data within the Tagbase environment. Significantly, it also allows for the automated reconciliation and harmonious visualization of both horizontal and vertical spatial tag data as linked map and profile plot displays. Such displays are central to analyses but difficult to achieve outside of Tagbase, particularly in the absence of a unified relational model for tagging data.

### NOAA ERDDAP data access

**Figure 6:**
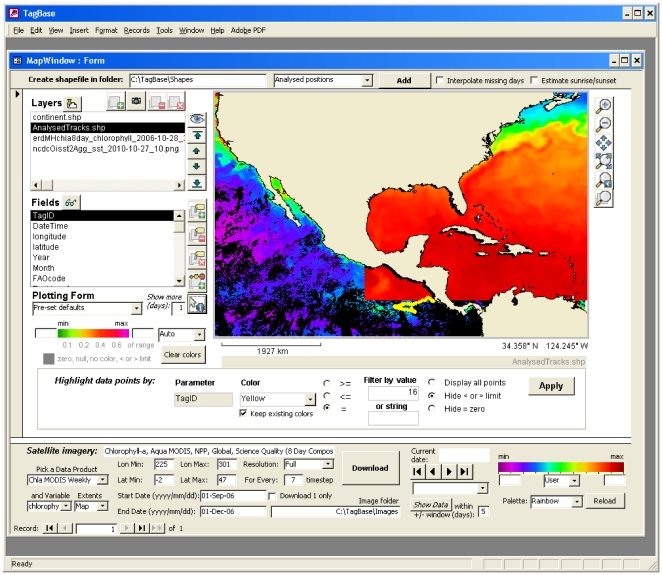
Displaying geographical information in Tagbase. Track point data from a striped marlin are displayed (yellow dots) along with overlays of satellite ocean color (NASA MODIS weekly chlorophyll-a) and sea-surface temperature imagery (NOAA ESRL Reynolds Optimum Interpolation) via Tagbase's integrated mapping form. Selected satellite imagery is downloaded on-the-fly from the NOAA ERDDAP website.

The potential of Tagbase's mapping component to integrate oceanographic information with tag data is extended by incorporating raster data layers from the NOAA ERDDAP catalogue (coastwatch.pfeg.noaa.gov/erddap/index.html). Specifically, any grid-based dataset, such as bathymetry, SST or sea-surface chlorophyll, hosted by the ERDDAP can be incorporated on-the-fly via a call to its web service. This offers a flexible means for integrating a diverse and extensive archive of oceanographic data products with Tagbase's mapping tool (Figure 6). Once an oceanographic image with date information is displayed on the map, displayed tag data can be filtered to show only those elements coincident with the time period of the image. This facilitates direct coupling between tag and oceanographic datasets, a typical requirement for tag research analyses rendered effortless within Tagbase.

### GIS Integration

Tagbase additionally provides mapping support by serving as a back-end database coupled dynamically to external GIS packages such as ArcGIS that support the ODBC protocol and SQL [36]. Via this mechanism, Tagbase has been previously interfaced with EASy GIS, a time dynamic mapping system for oceanographic applications used in marine biogeographic studies [37] and within which also the Fishtracker SST-matching geocorrection algorithm has been implemented [38].

### Results and Discussion

Electronic tagging studies have provided fundamental new insights into the behavior, physiology and spatial ecology of marine species. Both the increased accessibility of tagging technology and the utility of the information being yielded by this sampling platform for resource assessments [39], [40] has resulted in a proliferation of tag deployments. The fundamental conceptual challenge is one of ecological synthesis and quantitative analysis [41], [42], but for many researchers data management poses a significant practical constraint. There have been attempts to establish a centralized online repository for electronic tagging data [28], [43] and an institution-wide tag database for CSIRO [29]. However, such systems are not easily portable, and researchers typically lack the resources or data management expertise to implement them. Ultimately, it is the longer-term data legacy of tagging programs that may be at risk.

Tagbase was developed to address this critical need, and serves as an end-to-end tool for tagging applications. It is based on a comprehensive, extensible data model that supports a suite of tag manufacturer models in addition to deployment metadata and geolocation information. Tagbase is portable and scalable; it has been implemented on both small (Access) and enterprise-level data management platforms (SQL Server). Tagbase also includes a range of tools to facilitate bulk importation of diverse tag datasets, export to third party applications such as ODV [30] and geolocation routines [34], and connect dynamically to GIS software or other applications supporting ODBC connectivity [38]. Integral to Tagbase are a series of forms that provide standard reports of all tag data supported as plots or as tabular metadata via a simple to use graphical user interface. Such well-rounded functionality and its ease of use have resulted in the adoption of Tagbase by several groups running large electronic tagging programs on highly migratory species, including those at the Inter-American Tropical Tuna Commission, NOAA Southwest Fisheries Science Center, and University of Hawai'i at Mānoa.

Tagbase's development model emphasizes an open, community-based approach, with the Tagbase.org website serving as a focal point for development efforts, available tools and resources. Future development prioritizes on several areas: first is the porting of Tagbase to other widely used enterprise-strength database management systems and in particular non-proprietary, open-source systems like Postgre SQL. The intent here is to provide a greater range of options for users with extensive tag data collections, possibly constrained by budget or institutional database compliance requirements. Second is extending Tagbase support for remaining tag manufacturers and acoustic tag datasets. The third will be the development of browser-based client access through a series of web-forms that essentially reproduce the functionality of existing Tagbase forms. This will be useful particularly for larger, institutional user groups or tagging programs composed of a network of remote collaborators. The intent is to further facilitate interoperability and help ensure the accessibility and long-term legacy of tagging program data.

## References

[1] Humphries NE, Queiroz N, Dyer JRM, Pade NG, Musyl MK (2010). Environmental context explains Levy and Brownian movement patterns of marine predators.. Nature.

[2] Righton DA, Andersen KH, Neat F, Thorsteinsson V, Steingrund P (2010). Thermal niche of Atlantic cod Gadus morhua: limits, tolerance and optima.. Mar Ecol Prog Ser.

[3] Galuardi B, Royer F, Golet W, Logan J, Neilson J (2009). Complex migration routes of Atlantic bluefin tuna (Thunnus thynnus) question current population structure paradigm.. Can J Fish Aquat Sci.

[4] Howell EA, Hawn DR, Polovina JJ (2010). Spatiotemporal variability in bigeye tuna (Thunnus obesus) dive behavior in the central North Pacific Ocean.. Prog Oceanogr.

[5] Speed CW, Field IC, Meekan MG, Bradshaw CJA (2010). Complexities of coastal shark movements and their implications for management.. Mar Ecol Prog Ser.

[6] Hobday AJ, Flint N, Stone T, Gunn JS (2009). Electronic tagging data supporting flexible spatial management in an Australian longline fishery.. Tagging and Tracking of Marine Animals with Electronic Devices.

[7] Childers J, Snyder S, Kohin S (2011). Migration and behavior of juvenile north Pacific albacore (Thunnus alalunga).. Fish Oceanogr.

[8] Boustany AM, Matteson R, Castleton M, Farwell C, Block BA (2010). Movements of pacific bluefin tuna (Thunnus orientalis) in the Eastern North Pacific revealed with archival tags.. Prog Oceanogr.

[9] Schaefer KM, Fuller DW, Block BA (2009). Vertical movements and habitat utilization of skipjack (Katsuwonus pelamis), yellowfin (Thunnus albacares), and bigeye (Thunnus obesus) tunas in the equatorial Eastern Pacific Ocean, ascertained through archival tag data.. Tagging and Tracking of Marine Animals with Electronic Devices.

[10] Walli A, Teo SLH, Boustany A, Farwell CJ, Williams T (2009). Seasonal movements, aggregations and diving Behavior of Atlantic bluefin tuna (Thunnus thynnus) revealed with archival tags.. Plos One.

[11] Musyl MK, Brill RW, Curran DS, Fragoso NM, McNaughton LM (2011). Post-release survival, vertical and horizontal movements, and thermal habitats of five species of pelagic sharks in the central Pacific Ocean..

[12] Jorgensen SJ, Reeb CA, Chapple TK, Anderson S, Perle C (2009). Philopatry and migration of Pacific white sharks.. Proc R Soc B.

[13] Kraus R, Wells R, Rooker J (2011). Horizontal movements of Atlantic blue marlin (Makaira nigricans) in the Gulf of Mexico.. Mar Biol.

[14] Prince ED, Luo JG, Goodyear CP, Hoolihan JP, Snodgrass D (2010). Ocean scale hypoxia-based habitat compression of Atlantic istiophorid billfishes.. Fish Oceanogr.

[15] Hoolihan JP, Luo JG, Richardson DE, Snodgrass D, Orbesen ES (2009). Vertical movement rate estimates for Atlantic istiophorid billfishes derived from high-resolution pop-up satellite archival data.. Bull Mar Sci.

[16] Hays GC, Farquhar MR, Luschi P, Teo SLH, Thys TM (2009). Vertical niche overlap by two ocean giants with similar diets: Ocean sunfish and leatherback turtles.. J Exp Mar Biol Ecol.

[17] Shillinger GL, Palacios DM, Bailey H, Bograd SJ, Swithenbank AM (2008). Persistent leatherback turtle migrations present opportunities for conservation.. Plos Biol.

[18] Vetter R, Kohin S, Preti A, McClatchie S, Dewar H (2008). Predatory interactions and niche overlap between mako shark, Isurus oxyrinchus, and jumbo squid, Dosidicus gigas, in the California Current.. Calif Coop Ocean Fish Invest Rep.

[19] Kappes MA, Shaffer SA, Tremblay Y, Foley DG, Palacios DM (2010). Hawaiian albatrosses track interannual variability of marine habitats in the North Pacific.. Prog Oceanogr.

[20] Votier SC, Bearhop S, Witt MJ, Inger R, Thompson D (2010). Individual responses of seabirds to commercial fisheries revealed using GPS tracking, stable isotopes and vessel monitoring systems.. J Appl Ecol.

[21] Lehodey P, Senina I, Sibert J, Bopp L, Calmettes B (2010). Preliminary forecasts of Pacific bigeye tuna population trends under the A2 IPCC scenario.. Prog Oceanogr.

[22] McIntyre AD (2010). Life in the World's Oceans: Diversity, Distribution, and Abundance: Blackwell Publishing Ltd..

[23] Block BA, Costa DP, Boehlert GW, Kochevar RE (2002). Revealing pelagic habitat use: the Tagging of Pacific Pelagics program.. Oceanol Acta.

[24] Halpin PN, Read AJ, Best BD, Hyrenbach KD, Fujioka E (2006). OBIS-SEAMAP: developing a biogeographic research data commons for the ecological studies of marine mammals, seabirds, and sea turtles.. Mar Ecol Prog Ser.

[25] Urbano F, Cagnacci F, Calenge C, Dettki H, Cameron A (2010). Wildlife tracking data management: a new vision.. Philos Trans R Soc Lond B.

[26] Evans K, Arnold G, Nielsen JL, Arrizabalaga H, Fragoso N, Hobday A, Lutcavage M et al (2009). Summary Report of a Workshop on Geolocation Methods for Marine Animals.. Tagging and Tracking of Marine Animals with Electronic Devices.

[27] Costello MJ, Vanden Berghe E (2006). ‘Ocean biodiversity informatics’: a new era in marine biology research and management.. Mar Ecol Prog Ser.

[28] Coyne MS, Godley BJ (2005). Satellite Tracking and Analysis Tool (STAT): an integrated system for archiving, analyzing and mapping animal tracking data.. Mar Ecol Prog Ser.

[29] Hartog, Patterson TA, Hartmann K, Jumppanen P, Cooper S, Nielsen JL, Arrizabalaga H, Fragoso N, Hobday A, Lutcavage M et al (2009). Developing integrated database systems for the management of electronic tagging data.. Tagging and Tracking of Marine Animals with Electronic Devices.

[30] Schlitzer R (2002). Interactive analysis and visualization of geoscience data with Ocean Data View.. Comput Geosci.

[31] Sibert JR, Musyl MK, Brill RW (2003). Horizontal movements of bigeye tuna (Thunnus obesus) near Hawaii determined by Kalman filter analysis of archival tagging data.. Fish Oceanogr.

[32] Lam CH, Nielsen A, Sibert JR (2008). Improving light and temperature based geolocation by unscented Kalman filtering.. Fish Res.

[33] Nielsen A, Bigelow KA, Musyl MK, Sibert JR (2006). Improving light-based geolocation by including sea surface temperature.. Fish Oceanogr.

[34] Lam CH, Nielsen A, Sibert JR (2010). Incorporating sea-surface temperature to the light-based geolocation model TrackIt.. Mar Ecol Prog Ser.

[35] Nielsen A, Sibert JR (2007). State-space model for light-based tracking of marine animals.. Can J Fish Aquat Sci.

[36] Roberts JJ, Best BD, Dunn DC, Treml EA, Halpin PN (2010). Marine Geospatial Ecology Tools: An integrated framework for ecological geoprocessing with ArcGIS, Python, R, MATLAB, and C plus.. Environmental Modelling & Software.

[37] Tsontos VM, Kiefer DA (2002). The Gulf of Maine biogeographical information system project: developing a spatial data management framework in support of OBIS.. Oceanol Acta.

[38] Tsontos VM, O'Brien FJ, Domeier ML, Lam CH (2006). Description of an improved algorithm for automated archival tag geolocational estimation based on the matching of satellite SST and in situ temperature data: application to striped marlin (Tetrapturus audax) in the North Pacific.. ICES CM 2006/Q:.

[39] Taylor N, McAllister M, Block B, Lawson G (2009). A multi stock tag integrated age structured model for the assessment of Atlantic bluefin tuna.. SCRS/2008/097 SCRS/2008/.

[40] Bigelow KA, Maunder MN (2007). Does habitat or depth influence catch rates of pelagic species?. Can J Fish Aquat Sci.

[41] Kelling S, Hochachka WM, Fink D, Riedewald M, Caruana R (2009). Data-intensive science: A new paradigm for biodiversity studies.. Bioscience.

[42] Jones MB, Schildhauer MP, Reichman OJ, Bowers S (2006). The new bioinformatics: Integrating ecological data from the gene to the biosphere.. Annu Rev Ecol Syst.

[43] Sibert J, Ancheta J (2006). Electronic Tagging Database Repository User's Guide.. https://www.soest.hawaii.edu/tag-data/documentation/etdr/guidebook.html.

